# Supplementary material for: Jejunal Transcriptomic Profiling for Differences in Feed Conversion Ratio in Slow-Growing Chickens
Source: Animals (Basel). 2021 Sep 5;11(9):2606. doi: 10.3390/ani11092606 (PMC8470203; doi:10.3390/ani11092606)
Supplement: Supplementary file 1 [file animals-11-02606-s001.zip › animals-1339234-supplementary.pdf]

Table S1. Primers for RNA-seq validation through RT-qPCR.

Gene name	Gene ID	Accession no	Forward primer	Reverse primer	Product size (bp)	Efficiency
LY6E[a]	ENSGALG00000041621	NM_204775.1	AGTCCATCTCCAAAGGCTGC	TCACTGTTGGAAGCCTGC	349	104.39%
PLAC8	ENSGALG00000011190	NM_001389397.1	GGTTTCTGTTCTGGGCTGAGT	CGTCGTCTCTACGGCATCAG	504	98.15%
LOC771880	ENSGALG00000052285	NM_205235.1	AGCCACGTCAACAAGGACAT	ACGATGGTGACGATCAGAGC	149	95.99%
MLK[b]	ENSGALG00000002766	XM_015279230.2	CACCGATACCAGGAGCCAGT	AGCCAGCAAGTCCACGATCT	123	100.17%
AVD[c]	ENSGALG00000025945	NM_205320.1	GGCTCCAACATGACCATC	GGTGGACTCTGAAAACTTCC	193	100.29%
IFI6	ENSGALG00000013575	NM_001001296.5	GTCTGACCAGAACGTCCACA	TGCCTCACCAGACATGATCG	88	97.89%
PLA2G4B	ENSGALG00000008727	XM_015287124.2	GCTGGATGGCTTGTACTGGT	TCCAGCACAGTGTCTGCATATC	341	96.99%
LBFABP	ENSGALG00000004141	NM_204634.1	ACCCAGGCAGACTGTGACTAA	GCACACAAGCTTCCCATTTG	115	104.17%
GAPDH[*]	ENSGALG00000014442	NM_204305.1	GGTGGCCATCAATGATCCCT	CCGTTCTCAGCCTTGACAGT	105	106.76%

[a] The primers of *LY6E* were from Zhang et al. (2008) [1].

[b] The primers of *MLKL* were from Zhang et al. (2020) [2].

[c] The primers of *AVD* were from Dayabari et al. (2015) [3].

[*] *GAPDH* is a housekeeping gene.

References

1. Zhang, W.; Li, H.; Cheng, G.; Hu, S.; Li, Z.; Bi, D. Avian influenza virus infection induces differential expression of genes in chicken kidney. *Res. Vet. Sci.* **2008**, *84*, 374–381. https://doi.org/10.1016/j.rvsc.2007.05.015
2. Zhang, J.; Hao, X.; Xu, S. Selenium Prevents Lead-Induced Necroptosis by Restoring Antioxidant Functions and Blocking MAPK/NF-kappaB Pathway in Chicken Lymphocytes. *Biol. Trace. Elem. Res.* **2020**, *198*, 644–653. https://doi.org/10.1007/s12011-020-02094-y
3. Daryabari, H.; Akhlaghi, A.; Zamiri, M.J.; Pirsaraei, Z.A.; Mianji, G.R.; Deldar, H.; Eghbalian, A.N. Oral administration of supplementary biotin differentially influences the fertility rate and oviductal expression of avidin and avidin-related protein-2 in low- and high-fertility broiler line hens. *Poult. Sci.* **2015**, *94*, 289–295. https://doi.org/10.3382/ps/peu073

Table S2. Ensembl-derived GO terms associated with difference in FCR.

Category	GO ID	Term	*p*-value	Genes[1]
Enrichment of GO terms biological process (BP)	GO:0006749	glutathione metabolic process	0.0020	***MMACHC***, ***CHAC1***
	GO:0006950	response to stress	0.0052	***MLKL***, ***RASGRF1***, *PLAC8, OASL, TRIM27.2,* ***CHAC1***, *SPON2, LAG3, KNG1*
	GO:0006952	defense response	0.0031	***MLKL***, *PLAC8, OASL, TRIM27.2, SPON2, LAG3*
	GO:0007162	negative regulation of cell adhesion	0.0033	***MYOC***, *LAG3, KNG1*
	GO:0009605	response to external stimulus	0.0012	***MLKL***, *PLAC8, OASL, TRIM27.2, SPON2,* ***CDK5R1***, *LAG3, KNG1*
	GO:0009607	response to biotic stimulus	0.0003	***MLKL***, *PLAC8, OASL, TRIM27.2, SPON2, LAG3*
	GO:0009615	response to virus	0.0035	***MLKL***, *OASL, SPON2*
	GO:0021700	developmental maturation	0.0057	***MYOC***, ***C1QL1***, ***CDK5R1***
	GO:0035023	regulation of Rho protein signal transduction	0.0051	***RASGRF1***, ***MYOC***
	GO:0042551	neuron maturation	0.0017	***MYOC***, ***C1QL1***
	GO:0043207	response to external biotic stimulus	0.0002	***MLKL***, *PLAC8, OASL, TRIM27.2, SPON2, LAG3*
	GO:0044419	interspecies interaction between organisms	0.0002	***MLKL***, *PLAC8, OASL, TRIM27.2, SPON2, LAG3*
	GO:0048640	negative regulation of developmental growth	0.0082	*PLAC8*, ***CDK5R1***
	GO:0051607	defense response to virus	0.0012	***MLKL***, *OASL, SPON2*
	GO:0051707	response to other organism	0.0002	***MLKL***, *PLAC8, OASL, TRIM27.2, SPON2, LAG3*
	GO:0071695	anatomical structure maturation	0.0032	***MYOC***, ***C1QL1***, ***CDK5R1***
	GO:0098542	defense response to other organism	0.0002	***MLKL***, *PLAC8, OASL, TRIM27.2, SPON2, LAG3*
	GO:0005102	signaling receptor binding	0.0086	***RASGRF1***, ***MYOC***, ***CHAC1***, ***C1QL1***, ***CDK5R1***, *LAG3, KNG1*
Enrichment of GO terms molecular function (MF)	GO:0008138	protein tyrosine/serine/threonine phosphatase activity	0.0049	*PTPDC1*
	GO:0033218	amide binding	0.0063	***MMACHC***, *AVD,* ***LAPTM4B***
	GO:0035254	glutamate receptor binding	0.0007	***RASGRF1***, ***CDK5R1***

[1]Genes in ***bold*** represent those higher expressed in low-FCR jejunum.

Table S3. KEGG pathways associated with difference in FCR.

Pathway ID	Term	Number of genes	q-value	Genes[1]
ko04977	Vitamin digestion and absorption	1	0.0128	***MMACHC***
ko04370	VEGF signaling pathway	1	0.0223	***PLA2G4B***
ko00350	Tyrosine metabolism	1	0.0133	***LOC100857280***
ko04668	TNF signaling pathway	1	0.0124	***MLKL, MMP10***
ko04660	T cell receptor signaling pathway	2	0.0133	*LOC107049158, LOC771880*
ko05323	Rheumatoid arthritis	1	0.0252	***MMP10***
ko00830	Retinol metabolism	1	0.0135	***LOC100857280***
ko04014	Ras signaling pathway	2	0.0262	***PLA2G4B, RASGRF1***
ko05340	Primary immunodeficiency	2	0.0086	*LOC107049158, LOC771880*
ko04913	Ovarian steroidogenesis	1	0.0156	***PLA2G4B***
ko04217	Necroptosis	2	0.0135	***MLKL, PLA2G4B***
ko00980	Metabolism of xenobiotics by cytochrome P450	1	0.0135	***LOC100857280***
ko04730	Long−term depression	1	0.0219	***PLA2G4B***
ko00591	Linoleic acid metabolism	1	0.0133	***PLA2G4B***
ko04657	IL-17 signaling pathway	1	0.0252	***MMP10***
ko04640	Hematopoietic cell lineage	2	0.0124	*LOC107049158, LOC771880*
ko04912	GnRH signaling pathway	1	0.0300	***PLA2G4B***
ko00010	Glycolysis / Gluconeogenesis	1	0.0160	***LOC100857280***
ko00480	Glutathione metabolism	1	0.0135	***CHAC1***
ko04664	Fc epsilon RI signaling pathway	1	0.0239	***PLA2G4B***
ko00071	Fatty acid degradation	1	0.0133	***LOC100857280***
ko00565	Ether lipid metabolism	1	0.0137	***PLA2G4B***
ko00982	Drug metabolism - cytochrome P450	1	0.0133	***LOC100857280***
ko04610	Complement and coagulation cascades	1	0.0262	*KNG1*
ko05030	Cocaine addiction	1	0.0137	***CDK5R1***
ko05204	Chemical carcinogenesis	1	0.0154	***LOC100857280***
ko04514	Cell adhesion molecules (CAMs)	3	0.0086	*LOC107049158, ENSGALG00000051068, LOC771880*
ko00590	Arachidonic acid metabolism	1	0.0156	***PLA2G4B***

ko04612	Antigen processing and presentation	2	0.0124	*LOC107049158, LOC771880*
ko00592	alpha-Linolenic acid metabolism	1	0.0128	***PLA2G4B***

[1]Genes in ***bold*** represent those higher expressed in low-FCR jejunum.
